# Diet-Induced Microbiome’s Impact on Heart Failure: A Double-Edged Sword

**DOI:** 10.3390/nu15051223

**Published:** 2023-02-28

**Authors:** Alexandre Rodrigues, Alexandre Gonçalves, Juliana Morais, Ricardo Araujo, Inês Falcão-Pires

**Affiliations:** 1 INEB-Institute of Biomedical Engineering, University of Porto, 4200-135 Porto, Portugal; 2 i3S-Institute for Research & Innovation in Health, University of Porto, 4200-135 Porto, Portugal; 3 Department of Surgery and Physiology, Faculty of Medicine of the University of Porto, 4200-319 Porto, Portugal; 4 UnIC@RISE, Department of Surgery and Physiology, Faculty of Medicine of the University of Porto, 4200-319 Porto, Portugal; 5 Cintesis@RISE, Center for Health Technology and Services Research, 4200-450 Porto, Portugal

**Keywords:** heart failure, HFrEF, HFpEF, microbiome, inflammation, diet

## Abstract

Heart failure (HF) is a debilitating disease with a significant clinical and economic impact worldwide. Multiple factors seem to increase the risk of developing HF, such as hypertension, obesity and diabetes. Since chronic inflammation plays a significant role in HF pathophysiology and gut dysbiosis is associated with low-grade chronic inflammation, the risk of cardiovascular diseases is likely modulated by the gut microbiome (GM). Considerable progress has been made in HF management. However, there is a need to find new strategies to reduce mortality and increase the quality of life, mainly of HFpEF patients, since its prevalence continues to rise. Recent studies validate that lifestyle changes, such as diet modulation, represent a potential therapeutic approach to improve several cardiometabolic diseases, although their effects on the GM and its indirect cardiac impact still warrant further research. Hence, in this paper, we aim to clarify the link between HF and the human microbiome.

## 1. Epidemiology and Pathophysiology of Heart Failure

According to the American Heart Association, in 2018, 49% of adults suffer from cardiovascular disease (CVD), whose fatal event prevalence is as high as 3.68 events per 1.000 persons per year [1]. These statistics prove that CVD is the predominant cause of death worldwide, with a rising tendency. If left untreated, most CVD progress to heart failure (HF).

Heart failure is the final stage of many cardiac and vascular diseases [2]. HF is characterized by the heart’s inefficiency in properly pumping sufficient blood to meet the body’s requirements for nutrients and oxygen. Its dismal prognosis results in increased hospitalization and high mortality rates [3,4]. In 2017, 1–2% of European adults were living with HF, while in the USA, statistics from 2018 indicate a 4% prevalence that increases with age and affects more men than women [1,5]. In addition to ageing and sex, multiple other factors correlate with an increased risk of developing HF, such as hypertension, obesity, diabetes mellitus, systemic inflammation, smoking, dyslipidaemia, a sedentary lifestyle and dietary choices. In 2019, a study showed that patients with HF had a higher prevalence of noncardiac comorbidities such as hypertension, diabetes, liver disease and sleep apnoea which could lead to a worse prognosis and complications in disease management [6]. HF is classified into two subtypes according to patients’ ejection fraction: HF with Reduced Ejection Fraction (HFrEF) and HF with Preserved Ejection Fraction (HFpEF).

## 2. Heart Failure with Reduced Ejection Fraction

HFrEF patients present a variety of symptoms, including dyspnea, orthopnea, fatigue and ankle swelling, triggered by conditions such as myocardial infarction or idiopathic cardiomyopathy [7]. In these patients, myocardial remodelling includes left ventricular hypertrophy (LVH), extracellular matrix (ECM) changes, cardiomyocyte death and metabolic disorders [8]. In HFrEF, excessive wall stress triggers cardiac hypertrophy and activates fibroblasts, leading to increased synthesis of collagen, fibronectin and laminin and resulting in ECM remodelling [8]. The loss of cardiomyocytes via autophagy, apoptosis or necrosis is observed in post-myocardial infarction [9] or ischemic-injured patients [8]. Moreover, abnormalities in cardiac metabolism occur alongside LVH [8]. In healthy hearts, β-oxidation of fatty-acids provides about 60% of the total energy demand, while pyruvate oxidation, derived from glucose, provides the rest [3]. The cardiac metabolic shift found in HFrEF patients enhances glucose oxidation and the glycolytic pathway, promoting anaerobic metabolism [3] and oxidative stress due to increased reactive oxygen species (ROS) production [8]. Excessive ROS damages the mitochondria causing mitochondrial permeability, cell death [10] and deficient functioning of the cellular energetics machinery [8]. The depletion of myocardial energy reserve and mitochondrial dysfunction represents a major cause of dysfunction of the failing human heart [3]. 

The survival rate in HFrEF has improved over the last decades [11] as several interventions have effectively managed this HF subtype. HFrEF pharmacological therapies rely on inhibiting the renin-angiotensin-aldosterone and the sympathetic nervous systems [12,13], while other interventions include performing aerobic exercise and restricting sodium, smoking and alcohol intake [7]. Additionally, non-pharmacological therapies have proven highly effective, such as cardiac resynchronization therapy, implantable cardiac defibrillator, transcatheter mitral valve repair and wireless pulmonary artery pressure monitors, which seem to reduce HFrEF hospitalizations [7,14,15,16,17].

## 3. Heart Failure with Preserved Ejection Fraction

While considerable progress has been made in HFrEF management [18], therapeutic options for HFpEF remain limited and are coupled with poor prognosis and survival [19]. Thus, novel strategies are needed to reduce its morbimortality. Indeed, approximately 50% of HF patients have HFpEF and its hospitalization is surpassing HFrEF [20,21]. The rising incidence of HFpEF in Europe is closely associated with the ageing of the population [22,23] and linked to the increasing prevalence of cardiac and noncardiac comorbidities such as coronary artery disease [24], atrial fibrillation, hypertension, diabetes mellitus, chronic lung disease, anemia, cancer, hypothyroidism and obesity [19,25], but the link between them is multifactorial [19]. Notwithstanding, it has been proposed that the rising prevalence of HFpEF is, in part, associated with the increased awareness and diagnosis of this disease since the focus has been, until recently, on HFrEF [19]. 

Obesity is extremely common in the population and its prevalence has been rising to alarming levels over the past decades. A recent study showed that obesity and obesity-derived dysmetabolism, such as insulin resistance, hyperglycemia and hyperlipidemia, are strongly associated with HFpEF and its pathophysiology [21]. A possible explanation lies in the fact that obesity and its cardiometabolic factors (abdominal adiposity, dyslipidemia and insulin resistance) are responsible for a pro-inflammatory environment that triggers increased levels of IL-6, TNF-α, sST2 and Pentraxin 3 [26]. Additionally, nitric oxide unavailability and endothelial dysfunction possibly bolster systolic and diastolic dysfunction and left ventricular hypertrophy in HFpEF [26,27]. Lastly, obesity causes changes in vasculature and blood volume which, accompanied by the increased consumption of oxygen, results in ventricular hypertrophy, increased left ventricular diastolic pressure and mean pulmonary arterial pressure. Ultimately, all these pathophysiologic processes perpetuate the progression towards HFpEF [28]. 

The global pandemic of obesity has also resulted in the rise of metabolic syndrome, a group of biochemical and clinical abnormalities acknowledged as significant risk factors for heart disease. The prevalence of heart dysfunction is higher in patients with metabolic syndrome than in patients without this disease [29]. In the same way, the prevalence of diabetes mellitus, closely associated with left ventricular remodelling, increasing pressure and stiffness of the arterial walls, has been escalating over the past few years [30]. Diabetes and metabolic syndrome share several pathomechanisms, including insulin resistance, metabolic derangements, oxidative stress, endothelial and mitochondrial dysfunction that could ultimately result in HFpEF [31,32].

Since lifestyle choices, such as food and exercise, appear to have a significant role in CVD, it is reasonable to assume that modifying one’s lifestyle would have a major influence on preventing and treating HF patients.

## 4. Human Gut Microbiome

The development of high-throughput techniques and powerful analytic tools, such as next-generation sequencing (NGS) and omics, has permitted an accurate description of the spectrum of the microbial elements in the human gut, allowing comparisons between health and various disease states [33]. The human GM is a complex ecosystem consisting of organisms present in the human digestive tract comprising Bacteria, Archaea, and Eukarya domains [33,34]. The microbiome is interconnected with the host physiology and pathophysiology [35] and plays an important role in human health [36]. Considering the comparatively deeper understanding of the function of bacteria compared to the other microbiota members and, since the majority of these microorganisms reside in our gastrointestinal tract [37], bacteria from the GM will be the primary focus of this review.

Current knowledge suggests that the hosts establish a core microbiota at birth, beginning with intestinal colonization within a few hours after delivery and finishing at around four years of age [33]. Facultative anaerobes are the first colonizers creating an environment that promotes the colonization of strict anaerobes such as *Bacteroides*, *Clostridium* and *Bifidobacterium* [38]. The GM of neonates is characterized by low diversity, which is progressively colonized by Bacillota (formerly Firmicutes) and Bacteroidota (formerly Bacteroidetes) [38]. By the end of the first year of life, the infant possesses a very distinct microbial profile compared to the first weeks after birth [38]. The first acquisition of the GM depends on the delivery mode. Vaginally delivered babies are directly exposed to maternal faecal and vaginal microbes and have an abundance of Bacillota and Bacteroidota, especially *Lactobacillus* and *Prevotella*. In contrast, caesarean babies are more colonized with microbes from the skin, having delayed colonization of Bacteroidota, *Bifidobacterium* and *Lactobacillus* and being often colonized with *Clostridium* [39]. This information on the first colonizers is relevant because evidence suggests that obesity and its associated comorbidities, such as type 2 diabetes, CVD and apnea, are affected by the GM early in life [38]. An excessive weight gain during the first years of life results in lower levels of *Bifidobacterium* and higher levels of *Staphylococcus* and increases the risk of developing obesity [38].

The human GM in the large intestine is mainly comprised of an alliance between Bacillota and Bacteroidota, followed by Actinomycetota (formerly Actinobacteria), particularly *Bifidobacterium* [38]. In adults, the individual’s large intestinal GM reaches a stable climax community, consisting principally of Bacteroidota, Bacillota, Actinomycetota, and Pseudomonadota (formerly Proteobacteria) [40]. The ratio of Bacillota to Bacteroidota is considered to be important for gut health [33] and its stability may also be beneficial for the innate immune system [38]. The microbiota plays an important role in protecting the host from pathogenic species invasion by producing nonspecific fatty acids, peroxides, and highly specific bacteriocins that can inhibit or kill other harmful bacteria, and certain strains release proteases capable of deactivating bacterial toxins [37,41,42].

Even once a healthy person’s microbiome is fully established, dysbiosis or variations in microbial composition or diversity can occur due to dietary changes, antibiotic exposure, or illness [43]. Dysbiotic conditions can promote pathogenic species invasion and proliferation, as well as disturbed immune system, which maintains a balanced system of pro- and anti-inflammatory cytokines [43].

## 5. Human Gut Microbiome and Heart Failure

The gut microbiota regulates vascular inflammation and atherosclerosis by interfering with host metabolism via platelet and endothelial cell activation, which increases arterial thrombosis [44]. Additionally, the GM has been implicated in metabolic and low-grade chronic inflammation characteristics of HF, via many metabolites such as trimethylamine-N-oxide (TMAO), short-chain fatty acids and tryptophan, which are further explored in this review. In comparison to healthy control groups, studies revealed significant variations in the quantity and diversity of bacterial taxa in the gastrointestinal tract, which are related to diet-induced obesity, opening the door to exploring bacterial taxa as an individual predictor of cardiometabolic disease risk [44]. 

**Figure 1:**
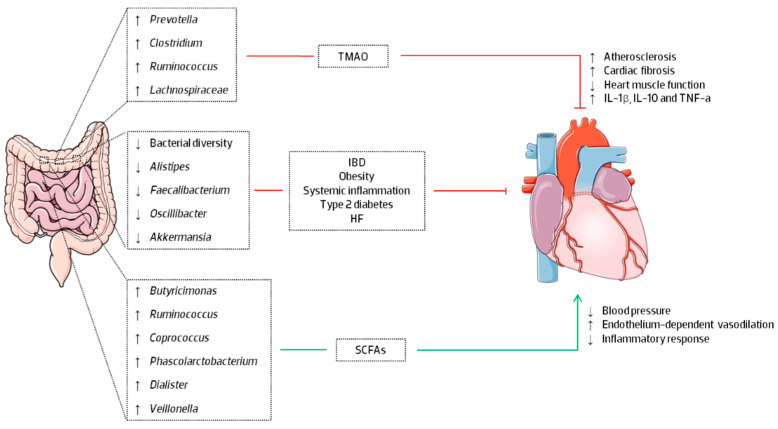
Schematic representation of the influence of gut microbiome (GM) modulation and metabolites in the cardiovascular system and in HF (heart failure). TMAO, trimethylamine N-oxide; SCFAs, short-chain fatty acids; IBD, inflammatory bowel disease; IL-1β, interleukin 1 beta; IL-10, interleukin 10; TNF-α, Tumor Necrosis Factor alfa; ↑, increased; ↓, decreased. The figure was produced using Servier Medical Art by Servier, which is licensed under a Creative Commons Attribution 3.0 Unported License (https://creativecommons.org/licenses/by/3.0/, accessed on 20 March 2022).

Recent studies have uncovered the association between microbiome changes and systemic inflammation, obesity, type 2 diabetes and cardiovascular diseases. The risk of HF is associated with alterations of GM (Figure 1), especially upon increased TMAO levels. Individuals with a higher abundance of the genera *Prevotella*, *Clostridium*, *Ruminococcus* and the family *Lachnospiraceae*, and lower levels of Bacteroidota demonstrated higher levels of plasma TMAO [45,46]. TMAO is an organic compound originated by GM’s digestion of dietary L-carnitine and phosphatidylcholine, mainly present in red meat and dairy products, into trimethylamine (TMA), which is converted into TMAO in the hepatocytes via flavin-monoxygenase-3 enzyme [47,48]. A study found that TMAO may modulate the metabolism of cholesterol and sterol, inhibiting reverse cholesterol transport and resulting in the development of atherosclerosis [45]. Furthermore, it has been demonstrated that TMAO increases myocardial stiffness by stimulating the transformation of fibroblasts into myofibroblasts, resulting in cardiac fibrosis and ECM remodelling [49,50]. TMAO also promotes the release of inflammatory cytokines such as TNF-α, IL-1β, and IL-10 and induces oxidative stress by decreasing endothelial nitric oxide synthase activity and nitric oxide [51]. In addition to the associations between TMAO and major adverse cardiovascular events and hypertension, a link has been established between all causes mortality, CVD mortality, diabetes mellitus, cancer, and renal function through the increase in renal fibrosis and dysfunction and a decrease in glomerular filtration rate [52].

Moreover, patients with pro-inflammation conditions, such as inflammatory bowel disease (IBD) and obesity, tend to have less bacterial diversity and fewer numbers of *Alistipes* (Bacteroidota), Faecalibacterium, Oscillibacter (Bacillota) and *Akkermansia* (Verrucomicrobiota) [53,54,55]. Indeed, the abundance of Bacteroidota increases as body weight decreases, but the abundance of *Clostridiaceae*, *Bifidobacteriaceae* and *Enterobacteriaceae* decreases [36,40]. Thus, microbiome modulation may prevent inflammation-induced deleterious cardiac consequences. For example, dietary intervention with *Akkermansia muciniphila* in obese patients improves obesity and metabolic parameters, such as insulin intolerance, higher plasma triglycerides and abdominal fat distribution [56]. In addition, supplementing the diet with *Lactobacillus* has been shown to alleviate obesity-associated metabolic complications by interacting with obesity-promoting bacteria and directly modulating the host immune system [57]. The specific supplementation with *Lactiplantibacillus plantarum* (formerly *Lactobacillus plantarum*) enhances endothelial-dependent vasodilation, NO bioavailability and lowers systemic levels of IL-8, IL-12 and leptin, cytokines linked with vascular inflammation and atherogenesis [58,59,60]. The observed reduction in leptin was related to a smaller myocardial infarct area and better post-infarction myocardial recovery since leptin stimulates the production of pro-inflammatory factors (IL-6, TNF-α, Th1 cells, and NK cells) that are known to contribute to vascular dysfunction and atherogenesis [58].

Excessive use of antibiotics eliminates or suppresses many components of the normal microbiota, setting the stage for pathogens’ growth and disease progression [37]. Antibiotic-associated diarrhea, particularly its most lethal manifestation, *Clostridium difficile* colitis, is a prime example of this dysregulation [37]. In certain cases, intestinal epithelial disruption permeates the gut wall, allowing for the bacteria from the gut to enter the submucosal compartments or even the systemic circulation, likely resulting in fatal sepsis [37], as in critically ill patients in the intensive care unit [37]. Although antibiotics lower pro-inflammatory cytokines and enhance endothelial function in HF patients, their use is quite controversial since they may result in the growth of drug-resistant microbes [61]. Therefore, more studies are needed to understand whether using antibiotics in specific conditions, such as HF, could improve heart function and survival rate. 

Short-chain fatty acids (SCFAs) are the main products of microbial fermentation activity in the gut. This fermentation is primarily carried out by *Butyricimonas* (Bacteroidota) and some Bacillota, such as *Ruminococcus*, *Coprococcus*, *Phascolarctobacterium succinatutens*, *Dialister* spp., and *Veillonella* spp. [54]. Recent research suggests that SCFAs are key cell signaling molecules that link gut microbial metabolism to blood pressure and vascular endothelial function via interactions with distinct G protein-coupled receptors. One example is the free fatty acid receptor 3 (FFAR3/GPR41) expressed in the sympathetic nervous system and the intestines. When activated, it reduces blood pressure and enhances endothelium-dependent vasodilation in mice, whereas GPR41 deficiency raises blood pressure and impairs endothelium-dependent vasodilation [62]. FFAR3 is also present in human cells, and activation of this receptor inhibits the inflammatory response in human monocytes [63]. The SCFAs acetate, butyrate, and propionate often associate under physiological situations to trigger important processes [64]. While butyrate promotes intestinal cAMP-induced gluconeogenesis, propionate directly activates the FFAR3 receptor and stimulates the glutamate-glutamine and GABA neuroglial cycles, boosting lactate production, thus suppressing hunger and nutrient intake [65,66]. Another GPCR expressed in adipose tissue, intestines, and immunological tissues is GPR43, which might operate as an energy sensor to stimulate other tissues to use the excess of energy rather than storing it as fat in adipose tissue, thereby maintaining metabolic homeostasis [67]. Therefore, SCFAs promote several physiological changes that likely improve cardiovascular and metabolic function in HF (Figure 1).

Heart Failure is characterized by mitochondrial dysfunction, which is one of the initial stages of metabolic remodelling in the failing heart [68]. Recent research has shown that the microbiota’s impact on tryptophan metabolism can have an effect on vascular inflammation, for example, indoxyl sulfate can induce oxidative stress, decrease NO production, and inhibit proliferation and wound healing [69]. Indole-3-propionic acid (IPA), which is mostly produced by *Clostridium sporogenes* in the gut, has been found to regulate mitochondrial function in supraphysiological levels by reducing maximum respiration and respiratory reserve capacity, two signs of cardiac remodelling that could eventually lead to HF [68]. Furthermore, cardiomyocytes treated with IPA showed decreased metabolic activity, fatty acid oxidation, and proliferation rates [68]. In contrast, it has been shown that IPA has protecting effects via reducing intestinal inflammation [69]. Curiously, the indole metabolism was found to promote serotonin release from gut cells and to amplify the inhibitory effects of serotonin on cells in the brain via the synthesis of tryptamine by the genera *Lactobacillus* and *Clostridium* [69]. The microbiota-derived tryptophan metabolite tryptamine appears to control serotonin release, which may influence the development of HF by contributing to vascular inflammation [69]. Nevertheless, clear mechanisms for these metabolites to act must still be established, which would be especially valuable for their eventual characterization.

According to various research, the diversity of GM is changed in HF. Patients with HFrEF, in particular, had a considerably reduced GM diversity index compared to controls, along with lower amounts of *Blautia*, *Collinsella*, unclassified *Erysipelotrichaceae*, and unclassified *Ruminococcaceae* [70]. Furthermore, patients diagnosed with HFrEF had greater levels of *Streptococcus* and *Veillonella*, as well as a decrease in SMB53 [71], whereas HFrEF patients on a low-fiber diet had a lower Bacillota/Bacteroidota ratio and increased intra-individual diversity [72]. In patients diagnosed with HFpEF, a depletion of bacteria that are SCFAs producers, namely *Ruminococcus,* was recently described [73]. Modulating the GM may be a therapeutic option for promoting weight loss in obese individuals, as well as weight gain in underweight people and overall health. The major approach requires taking specific probiotics, prebiotics, and/or symbiotic, which may have the potential to extend the beneficial effects of the GM significantly beyond local effects, resulting in beneficial cardiac effects, such as reducing inflammation and disease severity in diarrhea, IBD, colorectal cancer [74], which are all associated with pathogenesis and progression of HF. A pilot study has shown that taking probiotics can prevent cardiac failure and improve the quality of life in HF conditions [75]. However, more studies on this topic are urgently needed.

## 6. Human Gut Microbiome, Dietary Patterns, and Heart Failure

**Figure 2:**
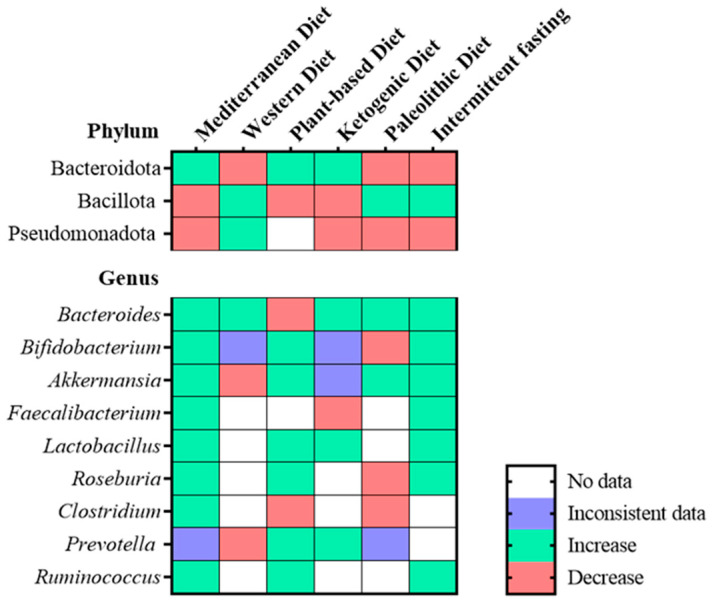
Heatmap representing the changes in the most prevalent bacteria found in the GM at the phylum and genus level in the Mediterranean diet, Western diet, plant-based diet, ketogenic diet, paleolithic diet and intermittent fasting [33,76,86,94,98,102,103,104,105,106,107,108].

Diet is the major player in the development and stability of the microbiome and its modulation is an opportunity to improve GM and its health benefits [76]. Dysbiosis has been linked to HF, and nutrition underlies GM’s homeostasis. Thus, understanding how nutrition and changes in the proportion of macronutrients impact GM is critical for determining its potential efficacy in HF prevention and overall health [38]. Six important dietary patterns and their influence on the GM have been addressed and the associated microbiota changes as depicted in Figure 2: Western diet (WD), Mediterranean diet (MD), plant-based diet (PBD), ketogenic diet, (KD), paleolithic diet (PD) and intermittent fasting (IF), since these diets have been implicated in health and disease.

The Western diet (WD) is marked by high sugar and refined carbohydrate intake with a high glycemic index, as well as a high-fat diet, red meat, preserved meat and fast food [77]. The high glucose and saturated fat content inhibits nitric oxide synthase, resulting in myocardial oxidative dysfunction, cardiomyocyte remodelling, and cardiac hypertrophy, all of which may predispose to HF [77]. This high-glucose and high fast-food diet causes dysbiosis by increasing Bacillota and Pseudomonadota, which raises the levels of ceramides and TMAO, further increasing cholesterol accumulation in macrophages and leading to atherosclerosis [77,78]. Furthermore, WD causes obesity, chronic inflammation, and lipid accumulation in the myocardium [79]. The possible negative effect of processed food on HF may be due to salt content and dietary additives, as processed meat includes significant quantities of sodium, which may raise HF risk through its influence on blood pressure. Moreover, high levels of dietary additives during fast-food production, such as nitrites have been linked to an elevated risk of HF and phosphate, which by affecting calcium phosphate metabolism, causes coronary heart disease and HF [79]. These additives and high-fat content alter the Firmicutes to Bacteroidetes ratio, especially by increasing the levels of *Erysipelotrichales*, *Bacilli*, and *Clostridiales* [80]. Furthermore, this diet promotes gut barrier permeability, which is described as leaky gut syndrome and caused mainly by the reduction in *Akkermansia muciniphila*, *Bifidobacterium* spp., *Bacteroidetes* spp., *Lactobacillus* spp., and *Clostridiales* spp., as well as all gut barrier-promoting bacteria. Moreover, an increase in *Oscillibacter* spp. and *Desulfovibrio* spp. microbes disrupt the intestinal wall integrity [80].

In contrast, the Mediterranean diet (MD) is regarded as the healthiest and most balanced dietary regimen compared to all diets. MD is rich in beneficial mono- and polyunsaturated fatty acids, polyphenols, antioxidants, fibers, and low glycemic carbohydrates, which increase the levels of SCFAs [57]. While no guidelines exist to encourage a specific diet to prevent HF, epidemiological studies have suggested that MD lower the risk and incidence of HF, highlighting the importance of consuming less saturated fat and more complex carbohydrates, fruits, and vegetables [77]. Moreover, some studies demonstrated that adhering to the MD improves obesity, lipid profile, and overall inflammation via the diet-derived growth of some bacteria [81,82]. The high production of SCFAs is associated with an increase in biodiversity, especially *Bacteroides*, *Lactobacillus*, *Bifidobacterium*, and *Ruminococcus* and a decrease in Bacillota and Pseudomonadota, all of which have a positive impact on human health and gut homeostasis by decreasing gut leakiness, enhancing immune function, improving insulin sensitivity, and thereby reducing inflammation, diabetes mellitus, and LDL, which are all recognized as risk factors for HF [83]. In addition, it has been shown that an 8-week intervention with MD in obese and overweight people resulted in increased levels of *Faecalibacterium prausnitzii*, *Roseburia*, and *Lachnospiraceae*, which are all fiber-degrading microbes, as well as overall increased gene richness, which is accompanied by a decrease in potential pro-inflammatory bacteria *Ruminococcus gnavus* and *Ruminococcus torques* [84]. Consequently, the microbiome changes were complemented by a decrease in LDL, HDL, and total plasma cholesterol, which are major risk factors of HF [84].

A plant-based diet (PBD) promotes the consumption of most plant-based meals while reducing or eliminating animal products [85]. It has been demonstrated that increasing plant-based food intake lowers systolic blood pressure and circulating triglyceride levels, preventing obesity and diabetes [85]. The consumption of nutrients with low bioavailability found in larger food particles, intact plant cell walls, and food without thermal treatment indicates that more nutrients reach lower levels in the gastrointestinal system, enhancing nutrient delivery to the gut microbiota [86]. This increases the production of microbial metabolites with various beneficial health effects, including local and systemic anti-inflammatory, anti-obesogenic, anti-hypertensive, hypocholesterolemia, and antioxidant effects [86]. SCFAs are increased in PBD and act as a substrate to maintain the intestinal barrier, which prevents endotoxemia and the subsequent inflammatory effects. Moreover, PBD-derived SCFAs increase thermogenesis, prevent obesity [86], and ameliorate diabetes mellitus and hypertension in metabolic syndrome patients, with all of these representing HF risk factors [87]. The vegetarian diet, a PBD that includes eating dairy products and eggs, changes the GM, especially by increasing two recognized butyrate-producing bacteria *Roseburia* and *Faecalibacterium* [88]. Furthermore, the vegetarian diet increases *Prevotella,* a fiber-degrading microbe, and decreases *Bacteroides*, an enterotype associated with protein and animal fat [88]. The GM changes result in the reduction in TNF-α and IL-10, which reduces inflammation and insulin resistance [88]. A more restrictive PBD diet is the vegan diet, which eliminates all animal products, which has been shown to alter the GM by decreasing pathogenic bacteria, such as *Enterobacteriaceae* and benefiting the microbial diversity by the increase in *Prevotella*, *Roseburia*, and *Ruminococcus*, bacteria that are currently considered to be protective against chronic inflammation, a risk factor of HF [86]. Together, the plant-based diet may be an efficient way to stimulate a diverse ecosystem of beneficial bacteria that support overall health.

The Ketogenic diet (KD) has a high content of fatty acids, low carbohydrate content, shares some pathways with the fasting state and some authors report a positive impact of ketones on HF [3,89]. After reducing carbohydrate intake, blood glucose levels decrease, resulting in glucose deficiency for the energy required by the body [89]. This energy demand could be provided by ketogenesis, a process that occurs mainly in the mitochondria of the hepatocytes resulting in the production of ketone bodies [90]. The beneficial effects of KD are possibly associated with changes in GM composition, in particular with the growth of beneficial bacteria, such as *Akkermansia muciniphila* and *Lactobacillus*, and the reduction in pro-inflammatory microbes, *Desulfovibrio* and *Turicibacter*, but overall the ketogenic diet decreased the microbial diversity [91]. A study found that KD decreased body weight and blood pressure in overweight patients while reducing total cholesterol, triglycerides levels and low-density lipoprotein cholesterol (LDL). Treating mice with ketones protected against myocardial injury by preserving cardiac function [91,92]. Ketogenic diets are often very restrictive, leading to poor patient compliance; therefore, supplementing the standard diet with ketone salts or esters could represent an alternative solution to achieve the results expected from ketogenesis without such a strict dietary plan. A recent study showed that ketone monoester drink intake increases ketones blood levels and decreases glucose tolerance and levels as soon as 30 min after its intake [93].

The Paleolithic diet (PD) is characterized by the consumption of vegetables, fruit, nuts, seeds, eggs, fish, and lean meat while eliminating grains, dairy products, salt, and refined sugar. PD has received a lot of attention in recent years due to its numerous potential health benefits [94]. The consumption of microbiota-accessible carbohydrates (MACs) from plant foods, as opposed to grains, processed foods, and foods containing refined sugars, as well as a higher intake of unsaturated fatty acids from olive oil, nuts and meat, may work synergistically to maintain cardiometabolic health [94]. PD shares many characteristics, such as a high intake of fruits, vegetables, fish, and nuts and a low glycemic load with MD [94]. Benefits of following a PD include weight loss and improved body composition and cardiometabolic indices such as HDL and LDL cholesterol, triglycerides and glucose levels, likely indicating a decrease in HF risk [95,96]. These metabolic improvements are accompanied by a more diverse GM ecosystem with an increase in the beneficial bacteria *Prevotella*, *Faecalibacterium*, *Ruminococcus*, *Lachnospira*, *Roseburia* and *Akkermansia* [94].

Intermittent fasting (IF) refers to eating habits in which individuals avoid caloric intake for periods lasting from 16 to 48 h, with intervals of regular food consumption in between [97]. The IF has been investigated as a viable therapy for reducing body weight and correcting negative metabolic parameters in obese and overweight people, all associated with an increased risk of HF. According to some research, IF lowers glucose, insulin, and leptin levels, which promotes insulin and leptin sensitivity; lowers resting heart rate, blood pressure, body fat, and inflammation [97]. Another study reported that IF increased the abundance of SCFAs, particularly acetate and butyrate in the faecal content of mice, indicating a decreased absorption of these SCFAs or increased fermentation of undigested carbohydrates since alternate-day fasting appears to increase the pyruvate fermentation pathway [98]. However, these findings remain unclear in humans since it has been reported that IF elevated SCFA-producing bacteria in the gut, but there was no substantial rise in circulating butyrate levels [98]. The time-limited regiment of IF causes a metabolic shift from glucose to fatty acids and ketones as fuel and alters the GM composition and function [99]. IF increases microbial diversity, especially *Akkermansia* and *Lactobacillus* and decreases the pathogenic bacteria *Alistipes* [99]. Nearly all levels of biological structure exhibit rhythmic patterns, which are regarded as one of life’s most crucial characteristics [99]. Many physiological functions, including metabolism and nutrition absorption, energy expenditure and immunological function, depend on host-microbiome circadian interactions. The diurnal bacterial localization through feeding is probably important in preserving the host’s capability to undertake a number of physiological processes, including nutrition intake and barrier integrity control [99].

Some of these popular dietary patterns have been investigated for their capacity to modulate intestinal microbiota. Dietary habits composed of non-refined foods and high consumption of MACs, such as MD, PBD, and PD influence the growth of microbes producing SCFAs, the main energy source and signal molecule between the host-microbe interactions and are believed to have a direct anti-inflammatory effect [57,100]. In contrast, the WD composed of high-fat sugars and low content in fibers reduces the production of SCFAs and expands the growth of bacteria associated with chronic inflammation, mainly Gram-negative producing lipopolysaccharides bacteria [101] and cancer-promoting nitrosamines [56].

## 7. Is There a Preferable Diet for HF Patients?

The Seven Country Studies was a landmark study that explored the relationship between CVD and lifestyle choices, particularly physical activity, diet fat composition, and blood cholesterol levels [109]. One of the findings was that dietary saturated fat increased serum cholesterol levels and CVD mortality [109]. Additionally, energy consumption and expenditure were crucial contributors to body fat and weight, which were closely related to blood cholesterol and linked to the development of coronary heart disease [110]. Moreover, major risk factors, including age, blood pressure, smoking habits, and serum cholesterol, could predict CVD death and all-cause mortality [111].

However, more recently, some controversial studies suggested no evidence between high saturated fat intake and increased CVD risk [112]. Furthermore, the quality and source of saturated fat had different implications on cholesterol levels and, therefore, in HF. For instance, polyunsaturated fatty acids favorably influence cholesterol levels and replace fatty acids with high-glycemic index carbohydrates, resulting in a higher risk for HF [112]. Another study revealed that increasing the intake or supplementing with omega-3 polyunsaturated fatty acids, including eicosapentaenoic acid, docosahexaenoic acid, and alpha-linolenic acid reduces the risk for CVD [113]. Therefore, the overall quality of fat is important, and carbohydrates seem more harmful than fat in CVD, contrary to the common belief [109]. As a result, the fundamental question is: What is the optimal macronutrients’ composition of a diet intended to reduce the risk of HF?

The answer is complex, indicating that several simplistic theories advocated the inclusion or exclusion of a single macronutrient, which is crucial for achieving cardiovascular health; however, cardiovascular and nutritional research is moving away from these concepts. While total caloric intake still matters, mindfulness about diet and health is the best predictor of success. Nevertheless, for long-term cardiometabolic health maintenance, healthy food-based models, such as minimally processed, plant-derived foods including fruits, non-starchy vegetables, nuts and seeds are the most appropriate [114].

Considering the challenge imposed by obesity treatment, the main strategy is its prevention by promoting healthier lifestyles and nutritional choices. Growing evidence indicates that energy imbalance results from several upstream effects, particularly poor diet quality, such as refined grains, sugars, alcohol, sugary drinks, processed foods, industrial trans fats, and high sodium content foods [114]. Aside from energy balance, diet quality influences metabolic dysfunction, predisposes to adiposity and induces changes in the gut microbiome [114]. Gut microbiome changes have a significant health impact [115]. Regardless of the dietary pattern, supplementation with prebiotics and probiotics seems to have a better impact on GM and its metabolites, especially in the case of patients that underwent metabolic surgery. A study showed that probiotic supplements improved post-bariatric surgery outcomes by reducing bacterial overgrowth and increasing weight loss [116]. The use of prebiotics could result in an increased abundance of *Bifidobacterium* and a higher reduction in body weight, which reduces diastolic and systolic blood pressure [116]. Prebiotics can promote the growth of helpful bacteria, such as *Lactobacillus* and *Bifidobacterium*, resulting in improved glucose and insulin tolerance and reduced inflammation and body weight [117], all linked to better HF outcomes. In contrast, a study found that supplementation with *Saccharomyces boulardii* for 3 months had no significant effect on the left ventricular ejection fraction or microbiota diversity in a population with HF [118]. Considering the impact of probiotics and diet changes on health, GM modulation could represent a target for personalized therapies in HF. More research is needed since there is a lack of clinically recognized evaluations concerning the most effective therapy, and nutrition-related recommendations are missing from the existing HF guidelines.

Faecal microbiota transplantation (FMT) used to treat *Clostridium difficile* infections, IBD, and several gastrointestinal and metabolic disorders, is another promising strategy as it alleviates HF comorbidities [116]. Recent research found that FMT improved insulin sensitivity and the abundance of SCFA-producing bacteria in individuals with metabolic syndrome [119]. Another study on mice showed that FMT could transmit diet-induced TMAO since the recipients of FMT from atherosclerotic donors demonstrated enhanced atherosclerosis compared to recipients from atherosclerotic-resistant donors, only in the presence of a high choline diet [120]. The efficacy of FMT remains to be studied since it depends on the ability of the donor to provide the necessary taxa capable of restoring metabolic deficits; therefore, new studies are needed to understand the role of FMT in restoring gut balance and its contribution to HF [121].

## 8. Conclusions and Future Perspectives

Heart failure is the most rapidly growing cardiovascular disease, overloading the healthcare systems worldwide. In high-income nations, advancements in public health altered the demographics toward an aged population with a higher prevalence of chronic diseases. Ageing societies need to improve their chronic disease management and health outcomes to reduce further healthcare expenditures and overall mortality. Diet-induced gut microbiome modulation is a very promising non-pharmacologic therapeutic approach since it may be associated with improvements in HF comorbidities, such as obesity, type 2 diabetes, cholesterol levels, and both systolic and diastolic blood pressure. However, the full impact of GM in HF remains to be explored. Since GM is influenced primarily by diet, personalized nutrition can be used to impact host immunity and health. However, it can act the other way around since an imbalance in gut microbiota can negatively influence the host’s health. Accordingly, the human gut microbiome could be seen as a double-edged sword, and it is imperative to focus on better understanding the interactions between diseases and the GM and the best approach to modulate this ecosystem through diet and prebiotics/probiotics or symbiotics supplementation.
